# Correlation between physician assistant students’ performance score of history taking and physical exam documentation and scores of Graduate Record Examination, clinical year grade point average, and score of Physician Assistant National Certifying Exam in the United States

**DOI:** 10.3352/jeehp.2020.17.16

**Published:** 2020-05-27

**Authors:** Sara Lolar, Jamie McQueen, Sara Maher

**Affiliations:** 1 Physician Assistant Studies Program, Wayne State University, Detroit, MI, USA; 2 Department of Health Care Sciences, Wayne State University, Detroit, MI, USA; Hallym University, Korea

**Keywords:** Academic performance, Documentation, Physical examination, Physician assistants, United States

## Abstract

**Purpose:**

Learning to perform and document patient history taking and physical exam (H&P) entails a major component of the first year academic education of physician assistant (PA) students at Wayne State University, USA. The H&P is summative of multiple aspects of PA education, and students must master communication with patients and other health care providers. The objectives of this study were first, to determine if there was a correlation between scores on the Graduate Record Examination (GRE) component testing and scores on graded H&Ps. The second objective was to identify a correlation between proficiency with H&P documentation and academic and clinical year grade point average (GPA) and Physician Assistant National Certifying Exam (PANCE) score.

**Methods:**

Subjects included 147 PA students from Wayne State University from 2014–2016. PA students visited local hospitals or outpatient clinics during the academic year to perform and document patient H&Ps. Correlation between the H&P mean scores and GRE component scores, GPAs, and PANCE scores were analyzed.

**Results:**

The subjects were 26.5 years-old (+6.5) and 111 females (75.5%). There was no correlation between the GRE component score and the H&P mean score. The H&P score was positively correlated with GPA 1 (r=0.512, P<0.001), with GPA 2 (r=0.425, P<0.001) and with PANCE score (r=0.448, P<0.001).

**Conclusion:**

PA student skill with H&P documentation was positively related to academic performance score during PA school and achievement score on the PANCE at Wayne State University, USA.

## Introduction

### Background/rationale

The physician assistant (PA) profession is consistently ranked as one of the top 10 best jobs in the United States, and there is a steady increase in the number of applicants to PA programs [1]. It is increasingly essential for PA programs to admit only students who will be successful. To be able to practice as a PA, a student must graduate from an accredited PA program and pass the Physician Assistant National Certifying Exam (PANCE). Learning the art and science of documenting a patient’s history taking and physical exam (H&P) is a core competency for all PA students and integral to their success as a practicing PA. Formulation of a successful H&P requires the ability to organize disparate information from multiple sources and present the material succinctly and coherently. Students must master communication with patients and other health care providers. It requires a high level of critical thinking and medical knowledge. Difficulties in formulating an H&P might suggest a student who will struggle in other aspects of PA education and passing the PANCE. This knowledge would help faculty members to offer additional support and academic advising to the struggling student earlier.

The Graduate Record Examination (GRE) has been a standard criterion used in PA graduate school application for admission. It is required by 60% of PA programs [1]. The GRE is a generalist exam that measures verbal reasoning (GRE-V), quantitative reasoning (GRE-Q), and analytical writing (GRE-A). Its use is based on study results, which demonstrated higher GRE scores correlated with success in graduate school [2]. However, the usefulness is less clear when applied to healthcare professions, which require more specialized training not evaluated in the GRE. Mixed results were found in studies whose authors examined the GRE score association with success in healthcare-related graduate programs. Varying degrees of association were found between GRE scores, graduate school grade point average (GPA) and national certification scores for students in PA [3,4] and other graduate health care sciences programs, including physical therapy [5], occupational therapy [6] and doctoral nursing [7]. Most specifically, inconsistent and non-conclusive results were found when assessing for a relationship between GRE scores and success on the PANCE [8,9]. While the usefulness of the GRE in determining the overall academic and professional success of a PA student is unclear, GRE component testing scores might be useful in deciding aptitude in specific aspects of didactic and clinical training, such as clinical documentation competence.

### Objectives

The purpose of this study is twofold. The first objective is to determine if there is a correlation between scores on GRE component testing and scores on graded H&Ps. The second purpose is to determine if there is a correlation between the proficiency score of H&P documentation and academic and clinical year GPAs and PANCE score.

## Methods

### Ethics statement

This project received expedited approval from the institutional review board of Wayne State University (#095816B3E).

### Study design

It is a retrospective, observational pilot study from a single public university.

### Participants

Subjects included 3 consecutive cohorts of PA students from Wayne State University, USA, from 2014 to 2016 (n=147).

### Setting

PA student education at this institution consists of 2 distinct years. The first year is an academic year composed of 3 semesters. The second year is composed of clinical rotations and runs concurrently without semester breaks. During the first academic year, students visited a local hospital or outpatient clinic 3–4 times a semester and composed an H&P based on the encounter. It resulted in a total of 1,323 H&P documents created over the study period. The required content and academic focus changed during the year as the student’s education progressed. Initially, the document was focused on format, but later on proceeded to contents that were rich in critical thinking, including the culmination of a complete history and physical exam with an impression and plan.

### Data sources/measurement

Five to 6 faculty were randomly assigned H&P papers throughout the year, to avoid repeat grading of the same student. Faculty included clinical PAs who were alumni of the PA program. Extensive training and education were provided by the PA program faculty. Preexisting grading rubrics were used by faculty to grade the H&Ps to promote inter-rater reliability [10] (Supplement 1). Inter-rater reliability was assessed by dual grading of a series of H&P papers by 2 random graders and calculating an intraclass correlation coefficient (ICC). The ICC between graders was 0.857 (95% confidence interval, 0.756–0 .918), suggesting good reliability between graders.

Data were collected by one investigator (S.L.) and included basic demographics such as sex and age of the subjects. The data were obtained from the PA school applications, Wayne State University grade reports, and the National Commission for Certification of Physician Assistants.

### Quantitative variables

Data included the year of matriculation into the program, GRE-V, GRE-Q and GRE-A, individual grades for H&P semesters I, II and III, overall GPA for academic year 1 (GPA 1) and clinical year 2 (GPA 2), and the PANCE score of those who matriculated the program. The H&P scores were averaged to create an overall mean score for each student. Two subjects are yet to sit for the PANCE and were not included in calculations involving PANCE scores (n=145).

Possible scores for the component GRE are as follows: GRE-V, 130–170, in 1-point increments; GRE-Q, 130–170, in 1-point increments; and GRE-A, 1–6, in 0.5-point increments. The PANCE maximum score was 800, and the lowest reported score was 200. The maximum score on the H&P was 100.

### Statistical methods

All data were analyzed using IBM SPSS ver. 25.0 (IBM Corp., Armonk, NY, USA). An alpha level of 0.05 was selected for all analyses a priori. The variables were checked for assumptions of normality, linearity, and homoscedasticity. Pearson product-moment correlation coefficients were computed to assess a number of relationships with each of the 3 GRE scores. Each GRE score (GRE-V, GRE-Q, and GRE-A) was compared to GPA 1 (didactic year), GPA 2 (clinical year), H&P mean score, and PANCE exam score. The Bonferroni approach was used on the GRE comparisons to control for type I error across the 4 correlations, which resulted in a P-value of less than 0.0125 (0.05/4=0.0125) to achieve significance. Pearson product-moment correlations were also computed to assess the relationships between age and each GRE score (GRE-V, GRE-Q, and GRE-A), H&P mean score, and PANCE exam score. The same comparisons were conducted for the sex of the subjects. The Bonferroni approach was again used and resulted in a P-value of less than 0.001 (0.05/5=0.01). The independent samples t-tests were used to compare means between males and females, and an intraclass correlation coefficient was established between graders.

## Results

### Participants

Data were collected from a total of 147 subjects. The participants were primarily female (75.5%, n=111). The subjects had a mean age of 26.5 (±6.5 years) with a mean GPA 1 of 3.73 (+0.26), mean GPA 2 of 3.78 (+0.22) and a mean PANCE exam score of 495.5 (+77.42) (Table 1) (Dataset 1).

### Main results

The GRE component scores are presented in Table 2, and the H&P mean scores are presented in Table 3. The overall mean of the H&P scores was 91 (+5.0). Results of the correlational analyses comparing GRE scores to other variables are presented in Table 4.

Three correlations were significant at P<0.0125 level. GRE-Q was moderately associated with GPA 1 (r=0.30, P<0.001) and GPA 2 (r=0.35, P<0.001). The higher the GRE-Q score, the higher the GPA in both years 1 and 2. GRE-Q was also weakly associated with PANCE score (r=0.21, P=0.10). The higher the GRE-Q score, the higher the PANCE scores. There was no correlation found between GRE-Q and the H&P mean. No statistical correlation was found between the GRE-V or GRE-A with GPA 1, GPA 2, H&P mean, or PANCE score (Table 4).

Results of the correlations analyses comparing H&P means to 3 variables were presented in Table 5. H&P scores were found to be strongly correlated with GPA 1 (r=0.512, P<0.001) and moderately correlated with GPA 2 (r=0.425, P<0.001) and PANCE score (r=0.448, P<0.001). The higher the overall H&P score, the higher the PANCE score. The mean H&P score accounted for 20% of the variance in PANCE exam scores (R^2^=0.201).

Age was found to be moderately correlated with GRE-V scores (r=0.36, P<0.001). The older a subject was, the higher the score on the GRE-V. In contrast, age had a small negative correlation with H&P means (r=-0.27, P<0.001). The older a subject was, the lower the H&P mean score. Independent t-tests revealed females had a higher overall H&P mean (mean=91.8, standard error [SE]=0.35) compared to their male counterparts (mean=89.6, SE=0.63, t(145)=3.09, and P=0.003), which represented an effect size of d=0.59 (moderate effect). No other significant differences were found when comparing the sex of a participant to each section of the GRE (GRE-V, GRE-Q, and GRE-A) and the PANCE score.

## Discussion

These results suggest GRE component scores had no significant or very low correlation with the performance score of a PA student in H&P documentation. The authors noted some students struggled with the H&Ps during the academic first year. Of the 147 students, 18 scored below the standard deviation of the mean grade for the H&Ps. At this institution, the H&Ps entailed a major component of first year training. It was summative of multiple aspects of PA education and included concepts from the patient evaluation, clinical medicine, pharmacology, anatomy and pathophysiology coursework. Difficulties in formulating an H&P might suggest a student who will struggle in other aspects of PA education. This knowledge might help faculty members offer additional support to the student earlier. Unfortunately, the GRE component testing provides little insight into which students might struggle.

Of the 3 GRE component scores, only the GRE-Q was correlated with the outcomes measured. The GRE-Q correlation was moderate in magnitude with GPA 1 and GPA 2 and small in magnitude with PANCE scores. The remaining 2 GRE components offered no significant correlation with any of the outcomes measured. These results add to the growing body of literature which suggests GRE testing adds little value in predicting which students might excel in PA school. It has been suggested by some authors the GRE might even be a barrier to entrance into graduate school and discourage diversity [11].

The results suggest PA student skill with H&P documentation is related to academic success during PA school and achievement on the PANCE. Logically, the H&P scores would be strongly correlated with academic year 1 GPA, as H&P documentation is a component of the academic year GPA. A successful student with H&Ps will need to be adept at many other core competencies, including interpersonal and communication skills, patient care, and practice-based learning. These are all factors that would help a student excel during clinical rotations and may account for the correlation of H&P scores with the clinical year 2 GPA. Most interestingly, we found a moderate correlation between H&P success and PANCE scores, accounting for 20% of the variance in the PANCE score. This finding supports authors of other studies who concluded that student performance in foundational coursework, including anatomy, physiology, pharmacology, and patient assessment, was related to success on the PANCE [12,13]. Difficulties in formulating an H&P might suggest a student who will struggle in other aspects of PA education and passing the PANCE.

### Limitations

The results of this study are limited by the small sample size and that it included only a single institution. Although previous authors purported the correlation of GRE scores and graduate school success, the first use of the GRE should only be to identify which students are academically prepared for graduate-level study. The standardized test is not geared to identify desired outcomes, such as students’ overall achievement or success with specific aspects of graduate education. Therefore, the results of this study should be interpreted cautiously, and educators should not over-rely on GRE scores to predict student success.

### Conclusion

PA student skill with H&P documentation was positively related to academic success during PA school and achievement on the PANCE. It suggests that H&P documentation is a valuable teaching exercise for PA students.

## Figures and Tables

**Table 1.** Demographics, physician assistant school GPA and PANCE scores

Variable	Mean±standard deviation	95% confidence interval	Median
Age (yr)	26.5±6.5	25.4–27.6	24
GPA 1	3.73±0.26	3.69–3.77	3.81
GPA 2	3.78±0.22	3.74–3.82	3.88
PANCE (n=145)	495.5±77.4	483–508	488

GPA, grade point average; PANCE, Physician Assistant National Certifying Exam; GPA 1, GPA academic year 1; GPA 2, GPA clinical year 2.

**Table 2.** GRE component scores

Variable	Mean±standard deviation	95% confidence interval	Median
GRE-V	152.4±5.77	151–153	152
GRE-Q	151.5±4.94	151–152	152
GRE-A	4.14±0.49	4.06–4.22	4

GRE, Graduate Record Examination; V, verbal; Q, quantitative; A, analytical writing.

**Table 3.** History taking and physical examination scores

Variable	Mean±standard deviation	95% confidence interval	Median
H&P Sem 1	91±4.9	90.3–91.9	92
H&P Sem 2	92±4.6	90.8–92.2	92
H&P Sem 3	91±5.1	90.3–92.0	92
Overall	91±5.0	90.6–91.9	92

H&P, history taking and physical exam; Sem, semester.

**Table 4.** Correlation of GRE component scores with 4 variables

Variable	GPA 1	GPA 2	H&P	PANCE^a)^
GRE-V				
Pearson correlation	0.193	0.175	0.001	0.158
P-value^b)^	0.019	0.034	0.986	0.058
GRE-Q				
Pearson correlation	0.301	0.354	0.136	0.212
P-value^b)^	≤0.001	≤0.001	0.100	0.010
GRE-A				
Pearson correlation	0.131	0.139	0.101	0.075
P-value^b)^	0.115	0.093	0.224	0.367

GRE, Graduate Record Examination; GPA, grade point average; GPA 1, GPA academic year 1; GPA 2, GPA clinical year 2; H&P, history taking and physical exam; PANCE, Physician Assistant National Certifying Exam; V, verbal; Q, quantitative; A, analytical writing.

a) n=145.

b) Bonferroni adjustment required a P-value of 0.0125 (0.05/4=0.0125).

**Table 5.** Correlations between performance score of history taking and physical examination with GPA and PANCE

Mean H&P score	GPA 1	GPA 2	PANCE
Pearson correlation	0.512	0.425	0.448
P-value^a)^	≤0.001	≤0.001	≤0.001

GPA, grade point average; PANCE, Physician Assistant National Certifying Exam; H&P, history taking and physical exam; GPA 1, GPA academic year 1; GPA 2, GPA clinical year 2.

a) Bonferroni adjustment required a P-value of .017 (0.05/3=0.017).

## Footnotes

**Data availability** Data files are available from Harvard Dataverse: https://doi.org/10.7910/DVN/MDFUX2 Dataset 1. Raw data of the correlation between physician assistant students’ performance of history taking and scores of Graduate Record Examination, clinical year grade point average, and Physician Assistant National Certifying Exam in the United States.

## Supplementary materials

Data files are available from Harvard Dataverse: https://doi.org/10.7910/DVN/MDFUX2

Supplement 1. Example of a history taking and physical grading rubric.

Supplement 2. Audio recording of the abstract.
